# Improving resistance to lepidopteran pests and herbicide using Sanming dominant genic male sterile rice (*Oryza sativa* L.)

**DOI:** 10.3389/fpls.2024.1525620

**Published:** 2024-12-19

**Authors:** Zhen Li, Xu Liu, Hua Zhang, Pingbo Li, Fangyin Yao

**Affiliations:** Institute of Wetland Agriculture and Ecology, Shandong Academy of Agricultural Sciences, Jinan, China

**Keywords:** S-DGMS rice, lepidopteran pests, crystal toxin genes, imazethapyr, ALS, genetic improvement

## Abstract

In order to improve both resistance to lepidopteran pests and resistance to the herbicide imazethapyr in mainstay *japonica* varieties of the Huang-Huai rice region, Sanming dominant genic male sterile (S-DGMS) rice was used as a platform to facilitate the pyramiding of functional genes and the replacement of the genomic background. Twelve novel lines were developed, each carrying a crystal toxin gene conferring resistance to lepidopteran pests and the *ALS^627N^* allele conferring resistance to herbicide imazethapyr in the background of a mainstay *japonica* variety. The genomic background of the 12 novel lines was examined using 48 specified molecular markers, and each line carried less than two polymorphic markers relative to the corresponding mainstay variety. All 12 lines displayed high resistance to lepidopteran pests and the herbicide imazethapyr. The major agronomic traits of the 12 lines showed no difference relative to the responding mainstay variety when sprayed with pesticide. The popularization of the 12 *japonica* lines could reduce the use of pesticides and provide highly efficient control of weeds and weedy rice in the future, thus promoting the development of *japonica* rice production. Therefore, S-DGMS rice could be a powerful tool for the genetic improvement of target traits in rice.

## Introduction

Male sterility is a valuable genetic resource that provides powerful breeding tools for hybrid seed production and breeding processes. Several types of male sterility have been utilized in rice breeding, including cytoplasmic male sterility (CMS), environment-sensitive recessive genic male sterility (ES-RGMS), and dominant genic male sterility (DGMS). Among these, CMS and ES-RGMS are used in the three-line hybrid rice system and the two-line hybrid rice system, respectively, to produce commercial hybrid seeds (Chen and Liu, 2014). In contrast, DGMS rice is used in recurrent selection breeding. When DGMS rice is used as the maternal recipient, the ratio of resulting fertile to sterile progeny plants is nearly 1:1, and sterile plants carrying the desired traits can be further used as maternal recipients to improve the performance of other traits. The adoption of DGMS rice eliminates the need for hand emasculation, the first step in hybridization, which is highly time-consuming, labor-intensive, and economically costly in large breeding programs. Several types of DGMS rice have been reported, with Sanming DGMS (S-DGMS) rice being widely used for recurrent selection in breeding programs in China (Zhang et al., 2022). Thus, S-DGMS rice can be used to improve desired traits during the genetic improvement of rice varieties.

Rice leaf folder and rice stem borer are lepidopteran pests that can attack rice plants throughout their entire growth period. These pests have been included in the First-Class Crop Disease and Insect List in China for many years. To date, no rice accessions have been reported to show resistance to lepidopteran pests, and their prevention and control depend on the frequent application of pesticides. The development of pest-resistant rice varieties could be the most economical, effective, and environmentally friendly method for controlling pests. Due to the lack of endogenous genes conferring resistance to lepidopteran pests, it is not feasible to develop resistant varieties using the conventional breeding method. Crystal toxin genes from *Bacillus thuringiensis* encode crystal proteins that exhibit high insecticidal activity against lepidopteran pests but are safe for nontarget organisms, such as mammals and birds. Thus, crystal toxin genes have long been favored by researchers for developing insect-resistant crops (Koul, 2020). Several transgenic rice lines expressing a range of crystal proteins, including Cry1Ab/1Ac, Cry1C, and Cry2A, have been developed, demonstrating high resistance to lepidopteran pests and stable inheritance (Chen et al., 2005; Tang et al., 2006; Tu et al., 2000). Therefore, the crystal toxin genes and their rice carriers could be directly used to improve resistance to lepidopteran pests in other rice varieties.

Weeds and weedy rice are two serious biotic stresses during rice cultivation, causing significant losses to both the yield and quality of rice grain. While the application of chemical herbicides is an economical and effective method for weed control, it is not effective for controlling weedy rice. Weedy rice belongs to the same genus as cultivated rice, and some weedy rice strains are de-domesticated from commercial rice (Kanapeckas et al., 2016). Over the past two decades, the strategy combining herbicide-resistant rice with specific herbicides has achieved significant success in simultaneously controlling both weeds and weedy rice (Sudianto et al., 2013; Tan et al., 2005). Acetolactate synthase (ALS) is a critical enzyme involved in the biosynthesis of three branched-chain amino acids (leucine, isoleucine, and valine) in plants and is the target of many ALS-inhibiting herbicides, including sulfonylureas and imidazolinones. Nonsynonymous substitutions in the *ALS* gene can change the protein structure and may produce resistance to herbicides. To date, several *ALS* alleles carrying different nonsynonymous substitutions have been reported to be resistant to herbicides, including *ALS^179V^* (An et al., 2024), *ALS^548M^* (Chen et al., 2021), *ALS^627N^* (Fei et al., 2018), and *ALS^653D^* (Tan et al., 2005), etc. Therefore, the development of novel rice varieties carrying herbicide-resistant *ALS* alleles could play important roles in the control of weeds and weedy rice.

In this study, to facilitate the control of lepidopteran pests, weeds, and weedy rice, three crystal toxin genes and herbicide-resistant *ALS^627N^* allele were introduced into four mainstay *japonica* varieties in the Huang-Huai rice region: ‘Runnong 11’, ‘Huageng 5’, ‘Shengdao 22’, and ‘Wuyugeng 377’. S-DGMS rice was used as a platform to facilitate the pyramiding of functional genes, combining marker-assisted selection of crystal toxin genes with phenotypic selection for the *ALS^627N^* allele. Twelve novel *japonica* lines were developed, each carrying a crystal toxin gene and the *ALS^627N^* allele in the background of a mainstay variety, and showing less than two polymorphic SSR markers relative to the corresponding mainstay variety. All 12 lines exhibited high resistance to both lepidopteran pests and the herbicide imazethapyr.

## Materials and methods

### Rice materials

‘Jingeng 818’ is a temperate *japonica* variety carrying the *ALS^627N^* allele and is resistant to imazethapyr, an ALS-inhibiting herbicide. ‘Huahui 1’, ‘T1C-19’, and ‘T2A-1’ are transgenic lines carrying a single copy of the crystal toxin genes *cry1Ab/Ac*, *cry1C*, and *cry2A*, respectively, in the background of the *indica* variety ‘Minghui 63’. ‘Runnong 11’, ‘Huageng 5’, ‘Shengdao 22’, and ‘Wuyugeng 377’ are four mainstay temperate *japonica* varieties in the Huang-Huai rice region.

Temperate *japonica* S-DGMS lines carry the heterozygous alleles *SDGMS*/*sdgms* and produce stable dominant male sterility when crossed with male-fertile parents, resulting in a 1:1 segregation ratio of male sterile to fertile plants in the next generation (Xu et al., 2023).

### Development of novel *japonica* lines carrying crystal toxin genes and the allele *ALS^627N^*

**Figure 1:**
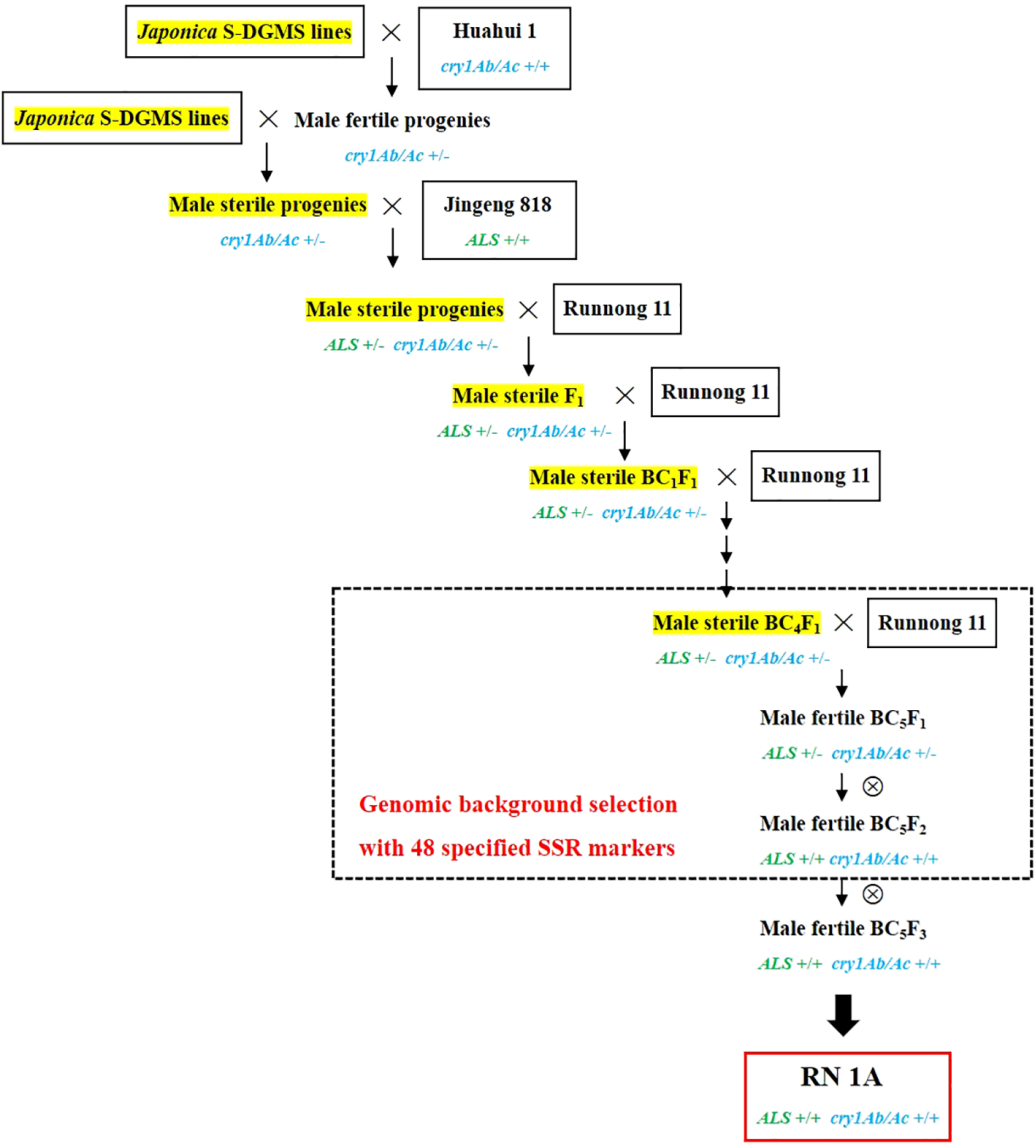
The development of ‘RN1A’, a novel *japonica* line carrying the *cry1Ab/Ac* gene and the *ALS^527N^* allele in the background of variety ‘Runnong 11’.

Take the development of a novel line carrying both *cry1Ab/Ac* and *ALS^627N^* in the background of ‘Runnong 11’ as an example. The detailed process is shown in **Figure 1**. First, a cross was made between a *japonica* S-DGMS sterile plant and ‘Huahui 1’, and two fertile plants carrying the *cry1Ab/Ac* gene were selected from 30 hybrid progenies. These two plants were then crossed with a *japonica* S-DGMS line to reduce the *indica* genomic background from ‘Huahui 1’. Subsequently, two male sterile plants carrying the *cry1Ab/Ac* gene from 30 hybrid progenies were selected to make the third cross with ‘Jingeng 818’. The resulting 100 hybrid progenies were subjected to phenotypic selection for the *ALS^627N^* allele and marker selection for the *cry1Ab/Ac* gene. Two sterile plants carrying both *ALS^627N^* and *cry1Ab/Ac* were then selected to make the fourth cross with the mainstay variety ‘Runnong 11’. Subsequently, a sterile F_1_ plant carrying both *ALS^627N^* and *cry1Ab/Ac* was selected to backcross with the recurrent male parent ‘Runnong 11’ for five generations. In each generation, phenotypic selection for the *ALS^627N^* allele and marker selection for the *cry1Ab/Ac* gene were conducted, and a sterile plant from 100 progenies was selected to backcross with ‘Runnong 11’. Considering the segregation of sterile and fertile plants, the free combination of the *ALS^627N^* allele and *cry1Ab/Ac* gene, and the germination percentage of hybrid seeds, at least 20 hybrid seeds should be produced from each cross or backcross to obtain a sterile plant carrying both *ALS^627N^* and *cry1Ab/Ac*. In addition, genomic background selection of male sterile plants was conducted in the BC_4_F_1_ generation, with the male sterile plant carrying both genes and the least number of polymorphic markers relative to ‘Runnong 11’ selected to backcross with ‘Runnong 11’. Next, at the BC_5_F_1_ generation, a male fertile plant carrying both genes and less than two polymorphic markers relative to ‘Runnong 11’ was selected to make a self-cross. At the BC_5_F_2_ generation, a total of 50 plants were subjected to selection for the two genes and genomic background, and five plants carrying homozygous *ALS^627N^* and *cry1Ab/Ac* and less than two polymorphic markers relative to ‘Runnong 11’ were selected to make a self-cross. Finally, one of the five BC_5_F_2_ plants was selected as the developed novel line and named as ‘RN1A’ (Runnong 11 carrying *cry1Ab/Ac* and *ALS^627N^*), with the BC_5_F_3_ progeny plants showing no segregation of major agronomic traits.

Similarly, the developed novel line carrying *cry1C* from parent ‘T1C-19’ and *ALS^627N^* in the background of ‘Runnong 11’ was named as ‘RN1C’, while the line carrying *cry2A* from parent ‘T2A-1’ and *ALS^627N^* in the background of ‘Runnong 11’ was named as ‘RN2A’. For the other three recurrent parents—’Huageng 5’, ‘Shengdao 22’, and ‘Wuyugeng 377’—the developed novel lines were named as ‘HG1A’, ‘HG1C’, ‘HG2A’, ‘SD1A’, ‘SD1C’, ‘SD2A’, ‘WYG1A’, ‘WYG1C’, and ‘WYG2A’, respectively, each carrying a crystal toxin gene and the *ALS^627N^* allele.

### Phenotypic selection of the *ALS^627N^* allele

The rice seedlings at the three- to four-leaf stage were uniformly sprayed with an imazethapyr solution at a dose of 150 g a.i. ha^−1^. Ten days after spray, the lines with green healthy leaves were regarded as resistant lines carrying the *ALS^627N^* allele and were then transplanted to paddy fields.

### Marker selection of crystal toxin genes

The three crystal toxin genes, *cry1Ab/Ac*, *cry1C*, and *cry2A*, were selected using the corresponding dominant markers, Cry1Ab/Ac, Cry1C, and Cry2A, respectively (Yang et al., 2011). The primer sequences for the three markers are listed in **Supplementary Table 1**. The PCR reaction and analysis of the PCR products for each marker were conducted as reported in a previous study (Li et al., 2023).

### Genomic background selection

According to “The protocol for identification of rice varieties-SSR marker method” (The Agricultural Industry Standards of China, NY/T 1433-2014), 48 specified SSR markers were used to distinguish between any two rice varieties (**Supplementary Table 2**). If a developed line carries two or more polymorphic markers relative to the recurrent parent, it is treated as a different line. If the number of polymorphic markers is one, the developed line is treated as a similar variety of the recurrent parent. If the number is 0, the developed line and the parental variety are considered the same. The similar or same variety could be directly put into commercial production, omitting the novel variety approval process.

The genomic background of the intermediate lines was examined using 48 specified SSR markers. In each generation, the line carrying the least number of polymorphic markers relative to the recurrent parent was selected for further backcrossing or self-crossing.

### Evaluation of resistance to herbicide, pests, and agronomic traits

The 12 developed lines and four recurrent parents were planted in 2021 and 2022 at the experimental farm of Yinmaquan in Jinan, China.

Seeds of all rice lines were sown in a seedling bed in early May. The rice seedlings at the three to four-leaf stage were uniformly sprayed with imazethapyr solution at a dose of 150 g a.i. ha^−1^. Herbicide resistance was evaluated 10 days after spraying. Lines with green healthy leaves were regarded as resistant lines, while those with yellow dead leaves were regarded as susceptible lines.

Seedlings of all rice lines were transplanted to paddy fields in mid-June. Two treatments were applied: one with pesticide spraying and the other without. In each treatment, the placement of rice lines followed a completely randomized block design with three replicates. Each replicate (plot) covered an area of 13.3 m^2^, with 16.67 cm between plants in a row and 25 cm between rows. For the treatment without pesticide, two indices were used to evaluate resistance to natural pest infestation: one was the percentage of plants with damaged leaves affected by rice leaf folders at the tillering stage, and the other was the percentage of white panicles caused by rice stem borers at the grain filling stage.

For the treatment with pesticide, agronomic traits of all rice lines were evaluated, including days to heading, plant height, number of panicles per plant, panicle length, number of grains per panicle, spikelet fertility, and 1,000-grain weight. The pesticide was applied four times during the growing season, at the seedling, tillering, heading, and filling stages, respectively, to prevent pest damage. In total, 20 plants were randomly selected from each replicate (plot), and the average value of agronomic traits was used for the value of each replicate. The evaluation of agronomic traits for each plant followed the method described in the book “Descriptors and Date Standard for Rice (*Oryza sativa* L.)” (Han et al., 2006).

### Data analysis

Raw data were processed using Microsoft Office Excel 2007. Statistical analysis was conducted with SPSS 22.0, and the LSD test was used to determine the significance of the differences between the mean values of each developed line and its recurrent parent. Figures were initially plotted using Microsoft Office PowerPoint 2007 and then processed using Adobe Photoshop CS6.

## Results

### Development of novel *japonica* lines

**Figure 2:**
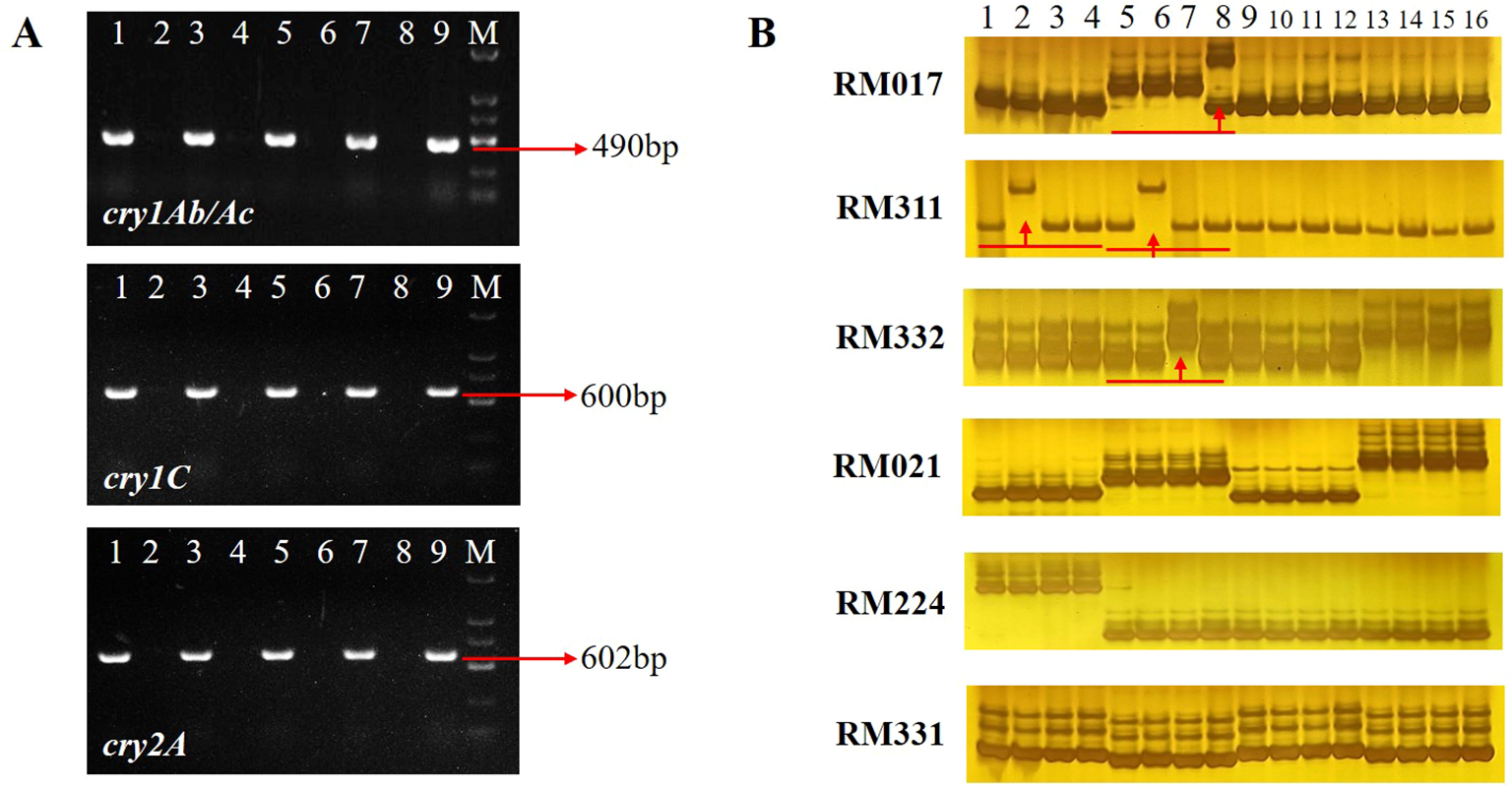
Marker-assisted selection and examination of novel developed lines. **(A)** Selection of crystal toxin genes. For *cry1Ab/Ac*, the numbers 1–9 indicate ‘Huahui 1’, ‘Runnong 11’, ‘RN1A’, ‘Huageng 5’, ‘HG1A’, ‘Shengdao 22’, ‘SD1A’, ‘Wuyugeng 377’, and ‘WYG1A’, respectively. For *cry1C*, the numbers 1–9 indicate ‘T1C-19’, ‘Runnong 11’, ‘RN1C’, ‘Huageng 5’, ‘HG1C’, ‘Shengdao 22’, ‘SD1C’, ‘Wuyugeng 377’, and ‘WYG1C’, respectively. For *cry2A*, the numbers 1–9 indicate ‘T2A-1’, ‘Runnong 11’, ‘RN2A’, ‘Huageng 5’, ‘HG2A’, ‘Shengdao 22’, ‘SD2A’, ‘Wuyugeng 377’, and ‘WYG2A’, respectively. M indicates the DL2000 DNA marker. **(B)** Examination of the genetic background of developed lines. The numbers 1–16 indicate ‘Runnong 11’, ‘RN1A’, ‘RN1C’, ‘RN2A’, ‘Huageng 5’, ‘HG1A’, ‘HG1C’, ‘HG2A’, ‘Shengdao 22’, ‘SD1A’, ‘SD1C’, ‘SD2A’, ‘Wuyugeng 377’, ‘WYG1A’, ‘WYG1C’, and ‘WYG2A’, respectively. Six markers showing polymorphism among the developed lines and recurrent parents (indicated by red lines and arrows) or polymorphism among the four recurrent parents are displayed, while the remaining 42 markers are shown in **Supplementary Figure 1**.

A total of 12 novel *japonica* lines were developed, each carrying a crystal toxin gene and the allele *ALS^627N^* in the background of a mainstay *japonica* variety (**Figure 1**; **Table 1**). Compared to the corresponding recurrent parents, four lines—namely ‘RN1A’, ‘HG1A’, ‘HG1C’, and ‘HG2A’—carried only one polymorphic site respectively, while the remaining lines showed the same background (**Figure 2**; **Supplementary Figure 1**). The information for all twelve novel lines is shown in **Table 1**.

**Table 1:** Detailed information of the 12 developed lines.

Name	Recurrent parent	Crystal toxin genes	*ALS* allele	Polymorphic site
RN1A	Runnong 11	*cry1Ab/Ac*	*ALS^627N^*	1
RN1C	Runnong 11	*cry1C*	*ALS^627N^*	0
RN2A	Runnong 11	*cry2A*	*ALS^627N^*	0
HG1A	Huageng 5	*cry1Ab/Ac*	*ALS^627N^*	1
HG1C	Huageng 5	*cry1C*	*ALS^627N^*	1
HG2A	Huageng 5	*cry2A*	*ALS^627N^*	1
SD1A	Shengdao 22	*cry1Ab/Ac*	*ALS^627N^*	0
SD1C	Shengdao 22	*cry1C*	*ALS^627N^*	0
SD2A	Shengdao 22	*cry2A*	*ALS^627N^*	0
WYG1A	Wuyugeng 377	*cry1Ab/Ac*	*ALS^627N^*	0
WYG1C	Wuyugeng 377	*cry1C*	*ALS^627N^*	0
WYG2A	Wuyugeng 377	*cry2A*	*ALS^627N^*	0

The polymorphic site indicates the number of polymorphic markers out of the specified 48 markers between each developed line and its recurrent parent, as shown displayed in **Figure 2** and **Supplementary Figure 1** in detail.

### Evaluation of resistance to imazethapyr

**Figure 3:**
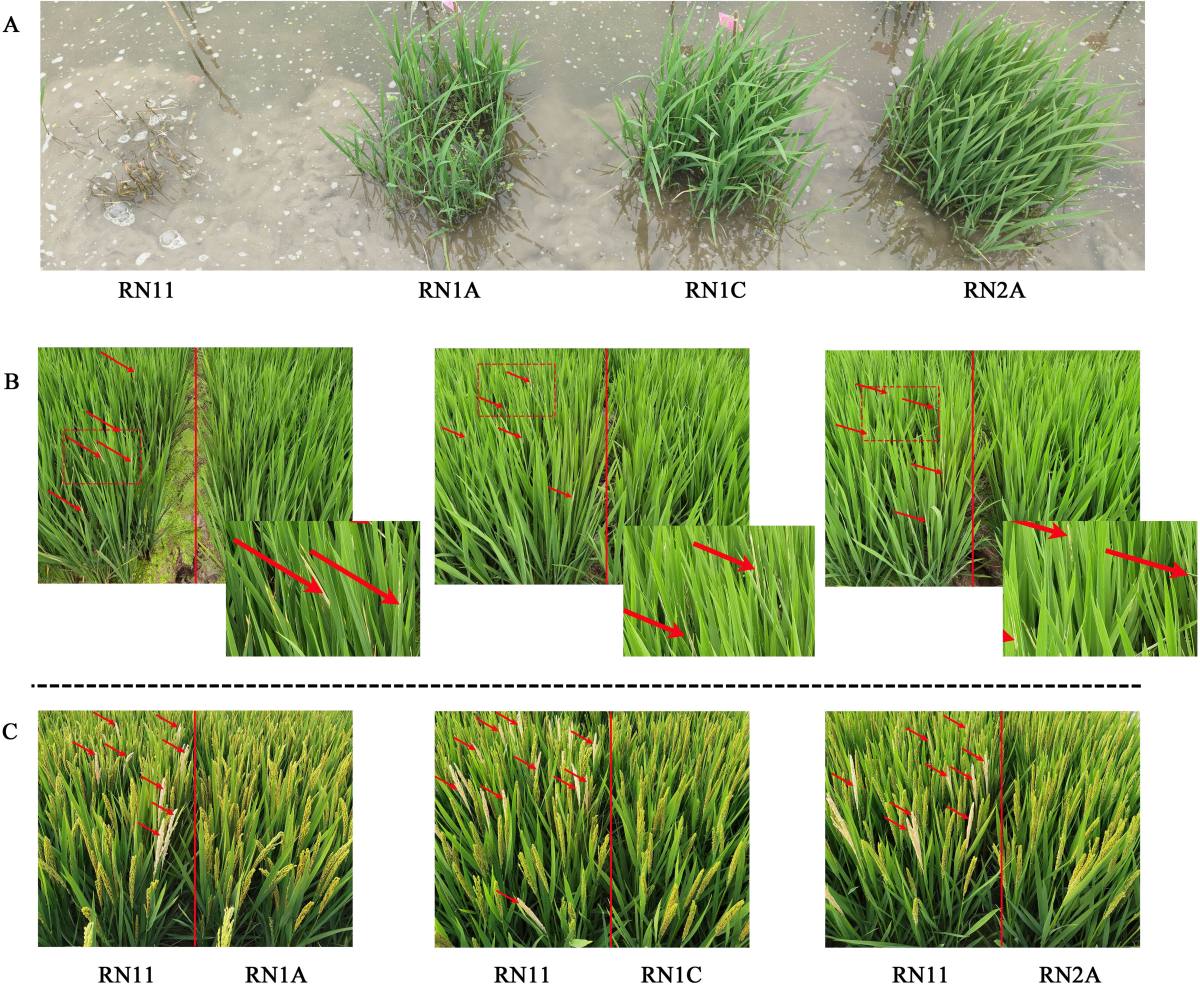
Resistance performance to imazethapyr **(A)**, leaf folders **(B)**, and stem borers **(C)** of the variety ‘Runnong 11’ and its three derived novel lines in the natural field. In **(B)**, red arrows indicate damaged leaves affected by leaf folders. The region surrounded by a red dashed line is enlarged and displayed in the lower right corner to better display pest infestations on leaves. In **(C)**, red arrows indicate white panicles affected by stem borers.

On the tenth day after spraying with imazethapyr solution, the four recurrent parents were shorter than the novel developed lines, with yellowed and withered leaves, indicating the death of the rice plants. In contrast, all 12 developed lines showed normal growth with healthy green leaves, indicating high resistance to imazethapyr (**Figure 3A**; **Supplementary Figure 2**).

### Evaluation of resistance to natural pest infestation

At tillering stage, the four recurrent parents were severely affected by leaf folders, with the percentage of plants affected reaching more than 90% in 2 years, indicating that nearly every plant of each parent had damaged leaves (**Supplementary Table 3**; **Figure 3B**; **Supplementary Figure 3**). In contrast, the 12 developed lines were only slightly affected by leaf folders, with values below 11%.

At grain filling stage, the four recurrent parents were attacked by stem borers, with the percentage of white panicles reaching more than 15% over 2 years (**Supplementary Table 3**; **Figure 3C**; **Supplementary Figure 4**). In contrast, the percentage for the 12 developed lines was less than 0.5% over 2 years.

### Agronomic performance in the field

The agronomic traits of the 12 developed lines and four recurrent parents were measured under pesticide treatment. The developed lines showed no significant differences in most traits compared to the corresponding parents (**Supplementary Table 4**; **Supplementary Figure 5**). Compared to the parent ‘RN11’, the line ‘RN1A’ headed approximately 2.67 days later in 2021, and the line ‘RN2A’ displayed lower values for panicle length in 2021 and for 1,000-grain weight in 2022. Compared to the parent ‘HG5’, the lines ‘HG1C’ and ‘HG2A’ headed approximately 4.33 and 4.67 days earlier in 2021, respectively. Compared to the parent ‘SD22’, the spikelet fertility of line ‘SD2A’ was about 4.81% lower in 2022. Compared to the parent ‘WYG377’, the line ‘WYG1C’ headed approximately 3.33 days earlier in 2021.

The grain yield per plot of the 12 developed lines and four recurrent parents was measured in two treatments: one with pesticide and the other without pesticide (**Supplementary Table 5**). The developed lines showed significantly higher grain yield per plot than the corresponding recurrent parents in the treatment without pesticide over 2 years, while showing no difference in the treatment with pesticide over 2 years.

## Discussion

In this study, the S-DGMS rice was used as a breeding platform to facilitate the pyramiding of crystal toxin genes and the *ALS^627N^* allele into the background of Huang-Huai mainstay *japonica* varieties, which greatly reduced the need for hand emasculation and simplified the crossing process. The target genes were introduced into S-DGMS rice in proper sequence, and the background of S-DGMS rice carrying target genes was gradually replaced with that of mainstay *japonica* varieties through a series of backcrosses, during which crystal toxin genes were selected using functional markers, and the *ALS^627N^* allele was selected with the herbicide imazethapyr. The developed lines showed high resistance to lepidopteran pests and the herbicide imazethapyr, and displayed nearly the same genetic background as corresponding mainstay varieties, proving the great power of S-DGMS rice in rice genetic improvement. The developed S-DGMS rice carrying the allele *ALS^627N^* could be further used as the maternal parent to make crosses with other rice materials carrying functional genes responsible for blast resistance, high yield, and superior quality, leading to the development of novel rice varieties exhibiting several desired traits. Thus, S-DGMS rice is a powerful tool in the recurrent selection of desired traits during the breeding process and will make significant contributions to the development of Green Super Rice varieties in the future (Zhang, 2008). Moreover, the developed 12 *japonica* lines could facilitate the reduced use of pesticides and highly efficient control of weeds and weedy rice in the future, thus promoting the development of *japonica* rice production in the Huang-Huai rice area.

## Data Availability

The original contributions presented in the study are included in the article/**Supplementary Material**. Further inquiries can be directed to the corresponding author.
