# Supplementary material for: Improving resistance to lepidopteran pests and herbicide using Sanming dominant genic male sterile rice (Oryza sativa L.)
Source: Front Plant Sci. 2024 Dec 19;15:1525620. doi: 10.3389/fpls.2024.1525620 (PMC11693452; doi:10.3389/fpls.2024.1525620)
Supplement: Supplementary file 1 [file Table1.docx]

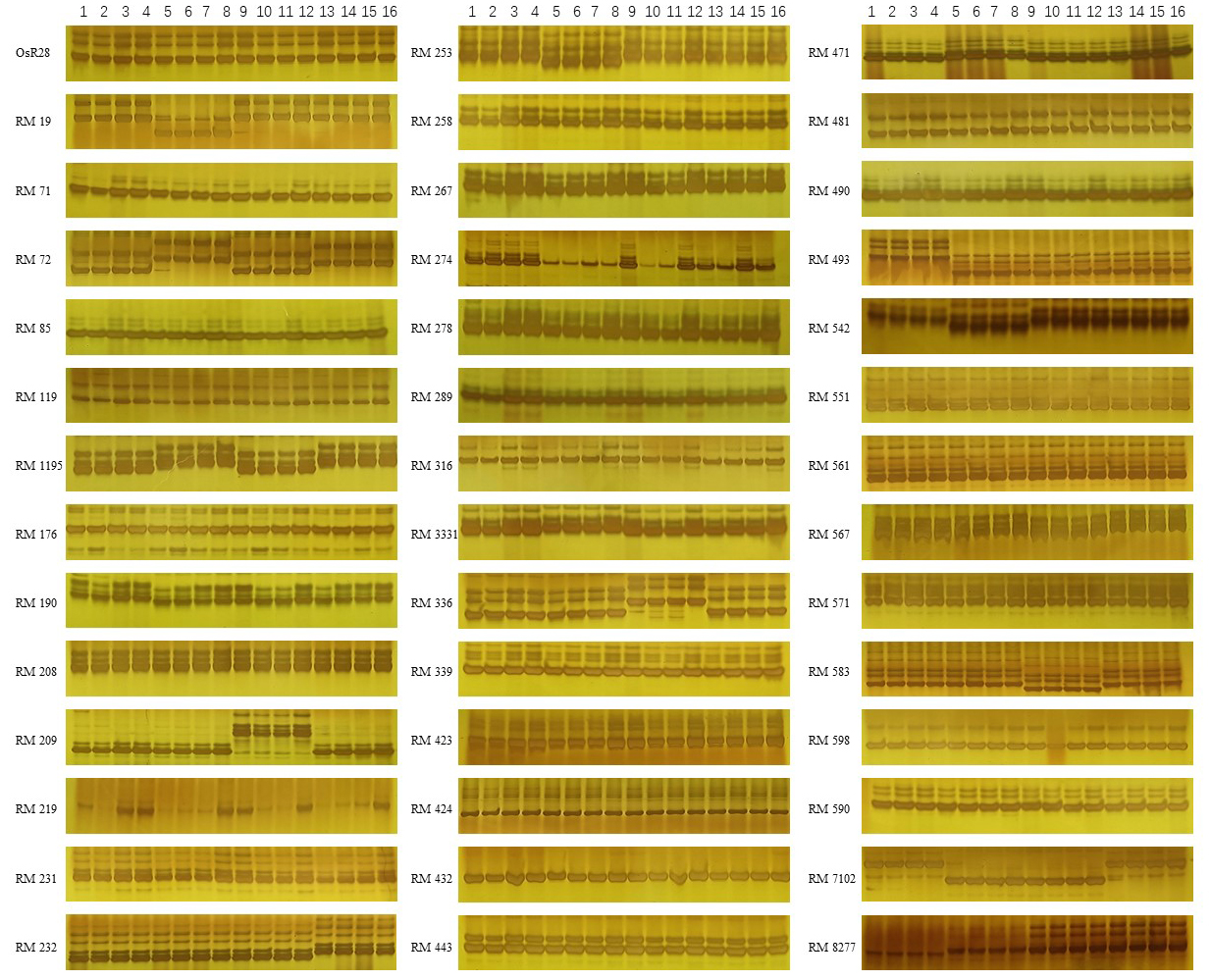


**Supplementary Fig. 1 Examination of genetic background of twelve developed lines with 42 specified SSR markers.**

The numbers 1-16 indicate ‘Runnong 11’, ‘RN1A’, ‘RN1C’, ‘RN2A’, ‘Huageng 5’, ‘HG1A’, ‘HG1C’, ‘HG2A’, ‘Shengdao 22’, ‘SD1A’, ‘SD1C’, ‘SD2A’, ‘Wuyugeng 377’, ‘WYG1A’, ‘WYG1C’, and ‘WYG2A’ respectively.


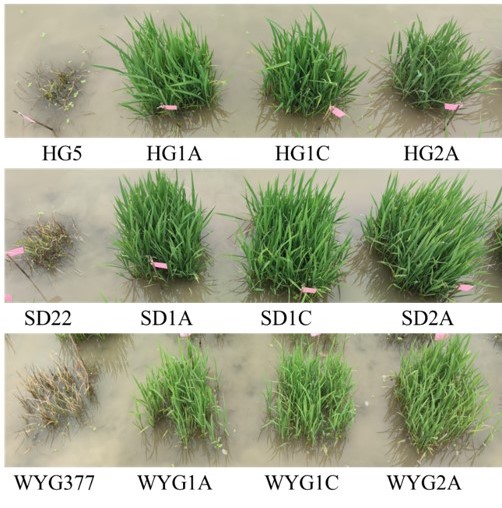


**Supplementary Fig. 2 Resistance performance to imazethapyr of three mainstay varieties and nine developed lines.**


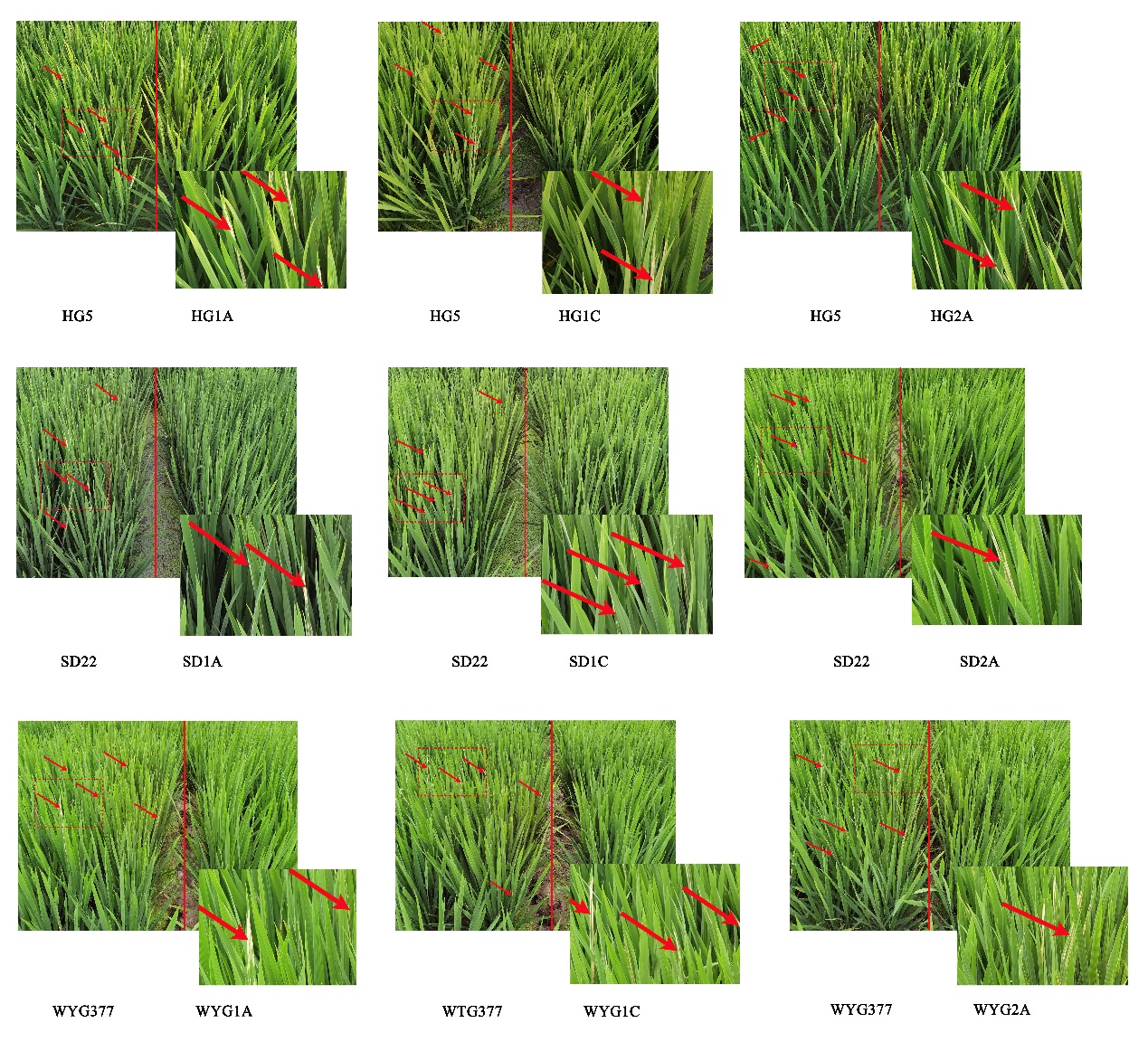
**Supplementary Fig. 3 Resistance performance to leaf folders of three mainstay varieties and nine developed lines in natural field.**

Red arrows indicate damaged leaves affected by leaf folders. The region surrounded with red dashed line was enlarged and put in the lower right corner, to better display pest infestations on leaves.


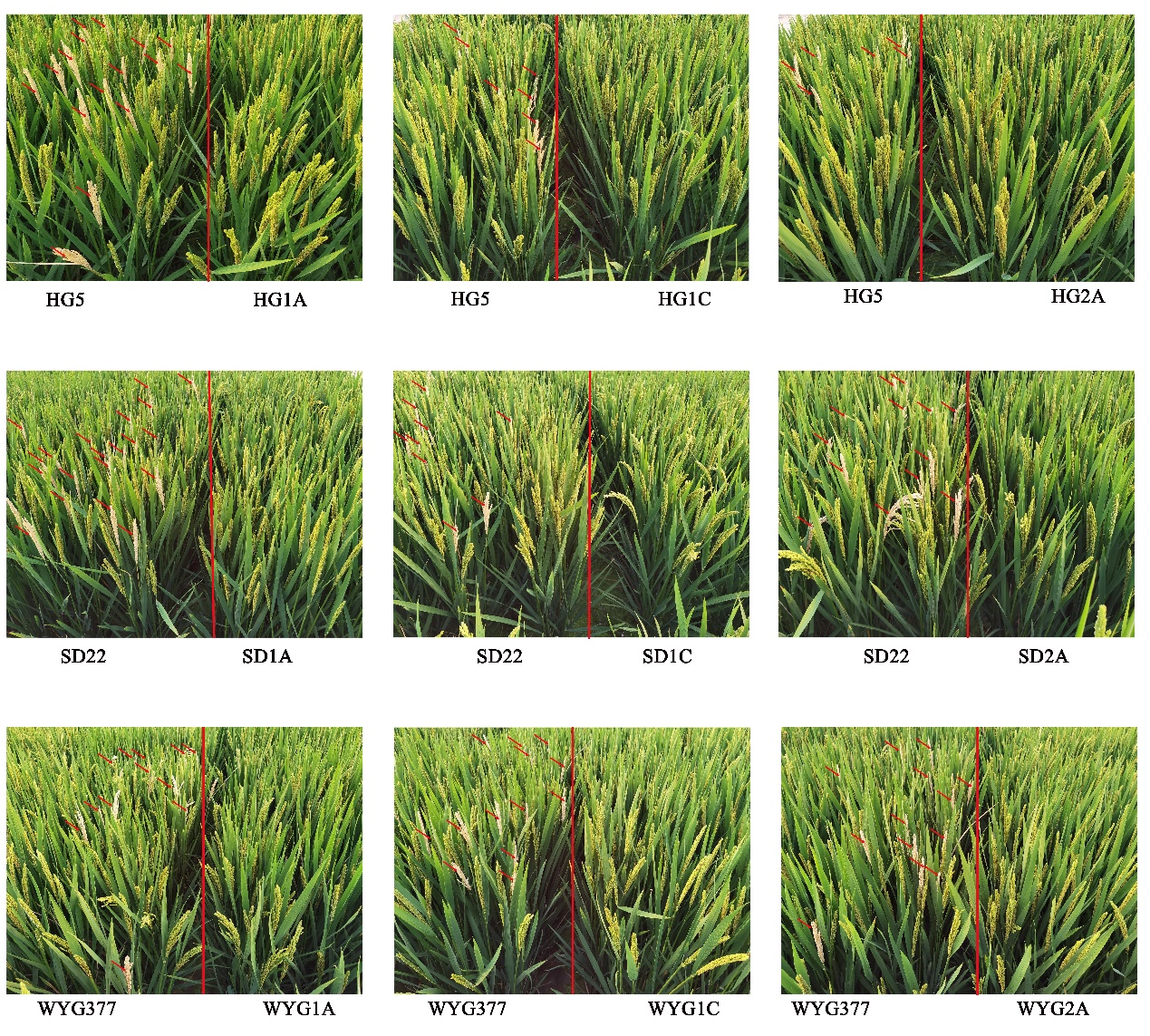
**Supplementary Fig. 4 Resistance performance to stem borers of three mainstay varieties and nine developed lines in natural field.**

Red arrows indicate white panicles affected by stem borers.


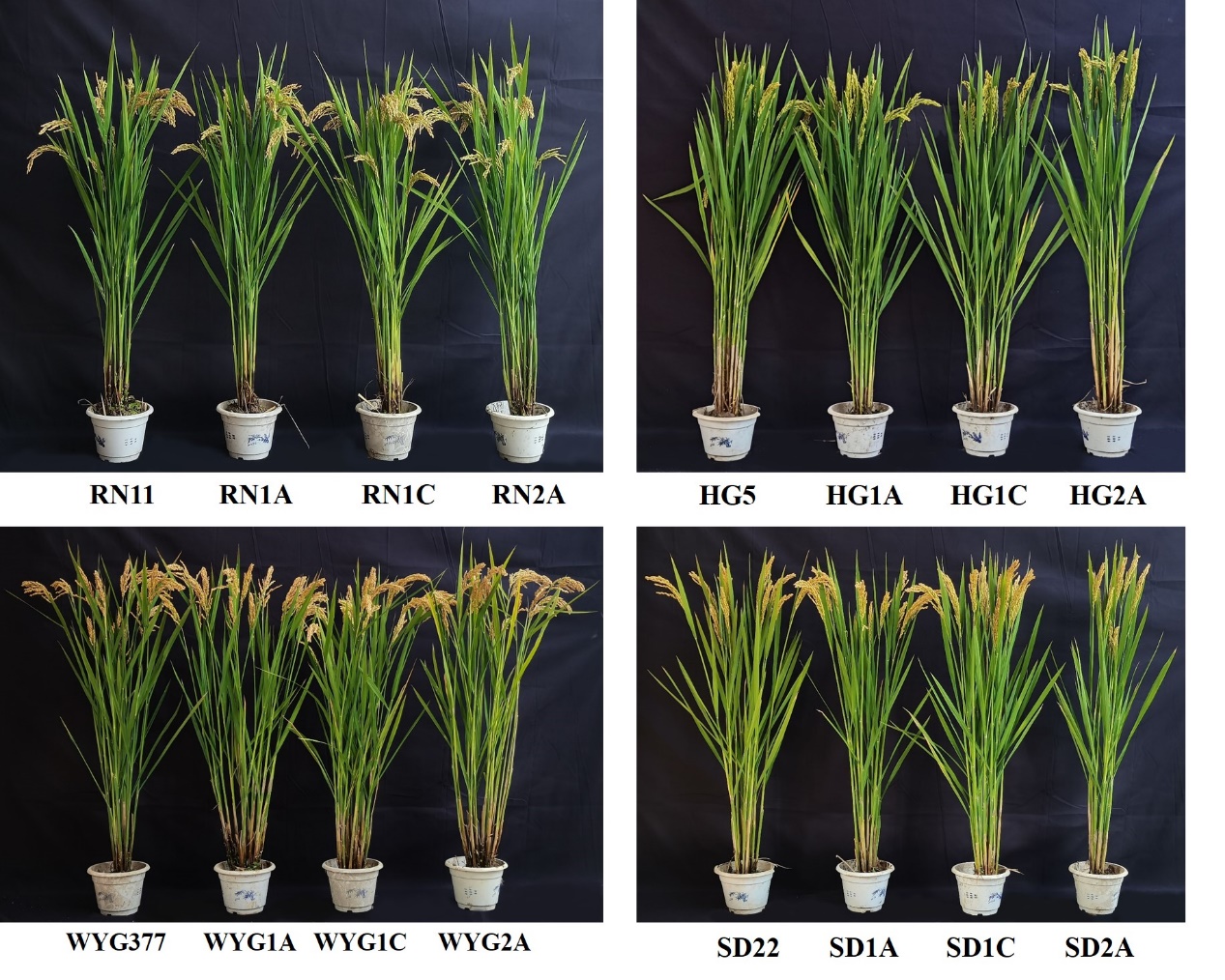


**Supplementary Fig. 5 Plant architecture of the four mainstay varieties and the twelve novel developed lines.**
