# Supplementary material for: Improving resistance to lepidopteran pests and herbicide using Sanming dominant genic male sterile rice (Oryza sativa L.)
Source: Front Plant Sci. 2024 Dec 19;15:1525620. doi: 10.3389/fpls.2024.1525620 (PMC11693452; doi:10.3389/fpls.2024.1525620)
Supplement: Supplementary file 2 [file Table2.docx]

**Supplementary Table 1 Molecular markers for selection of crystal toxin genes**

| Gene name | Primer name | Primer sequence(5’-3’) | Length(bp) |
| --- | --- | --- | --- |
| *Cry1Ab/Ac* | Cry1Ab/Ac-F | TCGAGACGTTAGCGTGTTTG | 494 |
|  | Cry1Ab/Ac-R | GAGGAAAGGTAAACTCGGGC |  |
| *Cry1C* | Cry1C-F | TTCTACTGGGGAGGACATCG | 602 |
|  | Cry1C-R | CGGTATCTTTGGGTGATTGG |  |
| *Cry2A* | Cry2A-F | CGTGTCAATGCTGACCTGAT | 600 |
|  | Cry2A-R | GATGCCGGACAGGATGTAGT |  |

**Supplementary Table 2 Information of 48 specified markers used for examination of genetic background**

| Primer name | Chr. | Sequence（5‘-3’） | |
| --- | --- | --- | --- |
| RM583 | 1 | F:agatccatccctgtggagag; | R:gcgaactcgcgttgtaatc |
| RM71 | 2 | F:ctagaggcgaaaacgagatg; | R:gggtgggcgaggtaataatg |
| RM85 | 3 | F:ccaaagatgaaacctggattg; | R:gcacaaggtgagcagtcc |
| RM471 | 4 | F:acgcacaagcagatgatgag; | R:gggagaagacgaatgtttgc |
| RM274 | 5 | F:cctcgcttatgagagcttcg; | R:cttctccatcactcccatgg |
| RM190 | 6 | F:ctttgtctatctcaagacac; | R:ttgcagatgttcttcctgatg |
| RM336 | 7 | F:cttacagagaaacggcatcg; | R:gctggtttgtttcaggttcg |
| RM72 | 8 | F:ccggcgataaaacaatgag; | R:gcatcggtcctaactaaggg |
| RM219 | 9 | F:cgtcggatgatgtaaagcct; | R:catatcggcattcgcctg |
| RM311 | 10 | F:tggtagtataggtactaaacat; | R:tcctatacacatacaaacatac |
| RM209 | 11 | F:atatgagttgctgtcgtgcg; | R:caacttgcatcctcccctcc |
| RM19 | 12 | F:caaaaacagagcagatgac; | R:ctcaagatggacgccaaga |
| RM1195 | 1 | F:atggaccacaaacgaccttc; | R:cgactcccttgttcttctgg |
| RM208 | 2 | F:tctgcaagccttgtctgatg; | R:taagtcgatcattgtgtggacc |
| RM232 | 3 | F:ccggtatccttcgatattgc; | R:ccgacttttcctcctgacg |
| RM119 | 4 | F:catccccctgctgctgctgctg; | R:cgccggatgtgtgggactagcg |
| RM267 | 5 | F:tgcagacatagagaaggaagtg; | R:agcaacagcacaacttgatg |
| RM253 | 6 | F:tccttcaagagtgcaaaacc; | R:gcattgtcatgtcgaagcc |
| RM481 | 7 | F:tagctagccgattgaatggc; | R:ctccacctcctatgttgttg |
| RM339 | 8 | F:gtaatcgatgctgtgggaag; | R:gagtcatgtgatagccgatatg |
| RM278 | 9 | F:gtagtgagcctaacaataatc; | R:tcaactcagcatctctgtcc |
| RM258 | 10 | F:tgctgtatgtagctcgcacc; | R:tggcctttaaagctgtcgc |
| RM224 | 11 | F:atcgatcgatcttcacgagg; | R:tgctataaaaggcattcggg |
| RM17 | 12 | F:tgccctgttattttcttctctc; | R:ggtgatcctttcccatttca |
| RM493 | 1 | F:tagctccaacaggatcgacc; | R:gtacgtaaacgcggaaggtg |
| RM561 | 2 | F:gagctgttttggactacggc; | R:gagtagctttctcccacccc |
| RM8277 | 3 | F:agcacaagtaggtgcatttc; | R:atttgcctgtgatgtaatagc |
| RM551 | 4 | F:agcccagactagcatgattg; | R:gaaggcgagaaggatcacag |
| RM598 | 5 | F:gaatcgcacacgtgatgaac; | R:atgcgactgatcggtactcc |
| RM176 | 6 | F:cggctcccgctacgacgtctcc; | R:agcgatgcgctggaagaggtgc |
| RM432 | 7 | F:ttctgtctcacgctggattg; | R:agctgcgtacgtgatgaatg |
| RM331 | 8 | F:gaaccagaggacaaaaatgc; | R:catcatacatttgcagccag |
| OSR28 | 9 | F:agcagctatagcttagctgg; | R:actgcacatgagcagagaca |
| RM590 | 10 | F:catctccgctctccatgc; | R:ggagttggggtcttgttcg |
| RM21 | 11 | F:acagtattccgtaggcacgg; | R:gctccatgagggtggtagag |
| RM3331 | 12 | F:cctcctccatgagctaatgc; | R:aggaggagcggatttctctc |
| RM443 | 1 | F:gatggttttcatcggctacg; | R:agtcccagaatgtcgtttcg |
| RM490 | 2 | F:atctgcacactgcaaacacc; | R:agcaagcagtgctttcagag |
| RM424 | 3 | F:tttgtggctcaccagttgag; | R:tggcgcattcatgtcatc |
| RM423 | 4 | F:agcacccatgccttatgttg; | R:cctttttcagtagccctccc |
| RM571 | 5 | F:ggaggtgaaagcgaatcatg; | R:cctgctgctctttcatcagc |
| RM231 | 6 | F:ccagattatttcctgaggtc; | R:cacttgcatagttctgcattg |
| RM567 | 7 | F:atcagggaaatcctgaaggg; | R:ggaaggagcaatcaccactg |
| RM289 | 8 | F:ttccatggcacacaagcc; | R:ctgtgcacgaacttccaaag |
| RM542 | 9 | F:tgaatcaagcccctcactac; | R:ctgcaacgagtaaggcagag |
| RM316 | 10 | F:ctagttgggcatacgatggc; | R:acgcttatatgttacgtcaac |
| RM332 | 11 | F:gcgaaggcgaaggtgaag; | R:catgagtgatctcactcaccc |
| RM7102 | 12 | F:taggagtgtttagagtgcca; | R:tcggtttgcttatacatcag |

Note: The markers in the table referred to ‘Protocol for identification of rice varieties-SSR marker method’ belonging to the agricultural industry standards of China (NY/T 1433-2014).

**Supplementary Table 3 Resistance performance of the developed lines and recurrent parents to natural pest infestation**

| **Line** | **Percentage of plants**  **affected by leaf folders** | | **Percentage of white panicles**  **affected by stem borers** | |
| --- | --- | --- | --- | --- |
|  | 2021 | 2022 | 2021 | 2022 |
| RN11 | 94.67±4.16 | 94.67±3.06 | 18.54±0.59 | 18.40±0.41 |
| RN1A | 2.00±2.00^**^ | 5.33±3.06^**^ | 0.25±0.10^**^ | 0.17±0.17^**^ |
| RN1C | 4.00±2.00^**^ | 3.33±1.15^**^ | 0.16±0.15^**^ | 0.27±0.09^**^ |
| RN2A | 9.33±3.06^**^ | 10.67±3.06^**^ | 0.25±0.22^**^ | 0.17±0.16^**^ |
| HG5 | 92.67±3.06 | 92.67±2.31 | 19.89±1.09 | 19.82±0.51 |
| HG1A | 4.00±2.00^**^ | 4.00±2.00^**^ | 0.20±0.08^**^ | 0.24±0.03^**^ |
| HG1C | 3.33±1.15^**^ | 2.67±1.15^**^ | 0.25±0.02^**^ | 0.19±0.07^**^ |
| HG2A | 2.00±2.00^**^ | 6.00±2.00^**^ | 0.23±0.08^**^ | 0.16±0.06^**^ |
| SD22 | 98.00±3.46 | 98.00±2.00 | 19.59±1.14 | 19.13±0.66 |
| SD1A | 1.33±1.15^**^ | 2.67±2.31^**^ | 0.24±0.08^**^ | 0.27±0.08^**^ |
| SD1C | 6.67±3.06^**^ | 5.33±1.15^**^ | 0.33±0.09^**^ | 0.31±0.02^**^ |
| SD2A | 4.00±2.00^**^ | 8.67±2.31^**^ | 0.27±0.11^**^ | 0.26±0.07^**^ |
| WYG377 | 97.33±1.15 | 99.33±1.15 | 16.65±0.32 | 16.60±0.81 |
| WYG1A | 2.00±2.00^**^ | 2.00±2.00^**^ | 0.21±0.01^**^ | 0.31±0.09^**^ |
| WYG1C | 2.00±2.00^**^ | 1.33±1.15^**^ | 0.32±0.08^**^ | 0.17±0.17^**^ |
| WYG2A | 2.67±3.06^**^ | 2.67±1.15^**^ | 0.27±0.11^**^ | 0.17±0.16^**^ |

Values are the means (± SD) (*n* = 3). **, significantly different from the recurrent parent at *P* < 0.01.

**Supplementary Table 4 Measurements of agronomic traits of the twelve developed lines and four recurrent parents in year 2021 and 2022**

| **Line** | **DTH (d)** | | **PH (cm)** | | **PNP** | | **PL (cm)** | | **NGP** | | **SF (%)** | | **GW (g)** | |
| --- | --- | --- | --- | --- | --- | --- | --- | --- | --- | --- | --- | --- | --- | --- |
|  | 2021 | 2022 | 2021 | 2022 | 2021 | 2022 | 2021 | 2022 | 2021 | 2022 | 2021 | 2022 | 2021 | 2022 |
| RN11 | 100.33±0.58 | 101.33±0.58 | 97.10±5.26 | 96.90±4.07 | 13.4±0.96 | 12.10±2.38 | 16.66±0.20 | 16.39±0.63 | 154.8±7.33 | 162.91±13.43 | 91.85±3.26 | 91.47±1.75 | 26.60±0.39 | 26.81±1.09 |
| RN1A | 103.00±1.00* | 103.67±1.53 | 98.10±4.15 | 96.80±4.52 | 12.0±0.94 | 12.10±2.13 | 16.37±0.25 | 16.30±1.31 | 157.9±8.14 | 175.93±15.94 | 90.50±3.33 | 92.48±1.88 | 26.06±0.30 | 26.54±1.03 |
| RN1C | 102.33±2.08 | 100.33±1.53 | 98.00±3.43 | 97.20±2.62 | 12.3±1.63 | 11.60±1.71 | 16.22±0.27 | 15.88±1.13 | 153.9±5.67 | 174.93±26.40 | 91.74±3.26 | 91.17±1.22 | 25.91±0.48 | 26.34±1.08 |
| RN2A | 101.00±1.00 | 100.67±1.53 | 98.80±3.77 | 97.10±4.70 | 11.4±0.69 | 12.30±2.71 | 15.88±0.20* | 16.63±1.07 | 163.0±3.71 | 165.19±21.70 | 91.43±3.09 | 91.17±2.02 | 25.99±0.39 | 25.77±1.15* |
| HG5 | 115.33±1.53 | 114.00±2.00 | 98.30±3.59 | 99.40±3.69 | 15.8±1.09 | 19.60±4.22 | 16.19±0.23 | 17.47±1.05 | 145.4±4.13 | 131.86±21.03 | 83.60±2.28 | 83.01±5.00 | 26.03±0.31 | 25.96±0.99 |
| HG1A | 116.00±2.00 | 114.67±2.52 | 98.30±3.65 | 98.80±4.57 | 19.5±2.13 | 18.20±3.71 | 17.09±0.41 | 17.67±1.20 | 152.7±7.78 | 120.23±10.79 | 83.13±6.19 | 84.84±2.69 | 25.97±0.44 | 25.40±0.95 |
| HG1C | 111.67±1.53* | 113.33±1.53 | 98.90±3.70 | 98.00±3.71 | 17.8±1.45 | 18.70±6.29 | 16.52±0.35 | 17.78±0.78 | 151.3±5.70 | 127.18±19.24 | 82.30±4.35 | 82.57±3.14 | 25.44±0.28 | 26.16±1.05 |
| HG2A | 111.33±1.15* | 113.67±1.53 | 99.40±3.13 | 99.80±2.74 | 15.9±1.97 | 17.80±4.24 | 17.09±0.28 | 17.75±1.09 | 158.9±4.81 | 124.53±24.05 | 84.69±2.44 | 85.32±2.95 | 25.10±0.30 | 26.14±0.75 |
| SD22 | 96.67±1.53 | 96.00±2.00 | 99.80±2.53 | 99.10±3.21 | 13.9±1.29 | 12.00±2.26 | 17.42±0.24 | 16.57±1.37 | 180.7±9.68 | 184.35±26.90 | 88.73±2.87 | 87.04±4.83 | 22.26±0.33 | 21.87±0.94 |
| SD1A | 95.33±1.53 | 94.00±2.00 | 101.30±2.79 | 99.70±2.95 | 12.1±1.09 | 13.20±3.91 | 17.43±0.36 | 16.83±1.00 | 174.3±9.74 | 161.16±28.45 | 87.84±3.96 | 86.92±4.91 | 21.52±0.26 | 21.86±0.88 |
| SD1C | 98.00±1.00 | 96.33±2.52 | 100.40±3.66 | 99.60±2.84 | 11.3±0.76 | 12.30±2.54 | 17.64±0.25 | 16.58±0.99 | 167.4±8.38 | 174.05±23.62 | 86.30±1.95 | 84.50±3.93 | 22.53±0.46 | 21.98±0.93 |
| SD2A | 99.00±1.00 | 98.33±1.53 | 101.10±3.00 | 101.50±3.06 | 9.9±0.60* | 13.40±3.50 | 18.23±0.29 | 16.87±1.14 | 183.0±8.82 | 174.24±32.17 | 88.03±4.24 | 82.23±2.15* | 21.60±0.37 | 22.21±0.95 |
| WYG377 | 104.33±1.53 | 102.33±2.08 | 100.10±4.84 | 101.10±2.33 | 11.1±1.12 | 11.30±3.53 | 18.31±0.28 | 18.40±0.77 | 174.4±7.06 | 176.08±26.93 | 90.40±2.49 | 89.45±2.91 | 25.33±0.16 | 25.70±1.28 |
| WYG1A | 104.00±1.00 | 101.67±1.53 | 99.80±4.64 | 100.30±2.75 | 10.2±0.74 | 11.10±2.13 | 18.10±0.24 | 18.32±0.93 | 181.4±5.52 | 176.77±23.08 | 88.75±3.58 | 90.32±1.81 | 24.63±0.50 | 25.32±1.41 |
| WYG1C | 101.00±1.00* | 102.33±1.53 | 102.00±4.47 | 100.80±3.71 | 11.1±0.84 | 10.80±2.30 | 17.62±0.41 | 18.33±0.66 | 173.2±9.19 | 178.34±20.00 | 87.88±3.21 | 90.23±1.50 | 24.87±0.20 | 26.37±0.89 |
| WYG2A | 103.33±1.53 | 104.67±0.58 | 99.90±2.08 | 100.60±2.32 | 9.9±0.72 | 12.30±2.26 | 18.10±0.18 | 18.71±0.54 | 184.9±7.79 | 167.53±21.84 | 87.72±3.06 | 90.34±1.49 | 25.17±0.27 | 25.34±1.56 |

DTH, days to heading. PH, plant height. PNP, panicle number per plant. PL, panicle length. NGP, number of grains per panicle. SF, spikelet fertility. GW, 1000-grain weight. *, significantly different from the recurrent parent at *P*<0.05, respectively.

**Supplementary** **Table 5 Grain yield per plot of the twelve developed lines and four recurrent parents in year 2021 and 2022**

| Line | 2021 (kg) | | 2022 (kg) | |
| --- | --- | --- | --- | --- |
|  | No pesticide | With pesticide | No pesticide | With pesticide |
| RN11 | 10.81±0.31 | 13.29±0.51 | 11.08±0.43 | 13.60±0.72 |
| RN1A | 12.43±0.19** | 13.12±0.18 | 12.42±0.24** | 13.32±1.03 |
| RN1C | 12.75±0.37** | 13.47±0.21 | 12.71±0.31** | 13.05±0.54 |
| RN2A | 12.12±0.03** | 13.26±0.26 | 12.28±0.21* | 13.02±0.50 |
| HG5 | 10.37±0.50 | 13.43±0.39 | 11.16±0.25 | 13.38±0.31 |
| HG1A | 12.15±0.18** | 13.27±0.54 | 12.40±0.11* | 13.28±0.82 |
| HG1C | 12.70±0.22** | 13.24±0.52 | 12.92±0.43* | 13.09±0.88 |
| HG2A | 12.70±0.37** | 13.21±0.34 | 12.92±0.77* | 13.48±0.42 |
| SD22 | 10.39±1.01 | 13.23±0.83 | 9.88±0.50 | 13.38±0.40 |
| SD1A | 12.93±0.39* | 13.54±0.43 | 12.71±0.35** | 13.25±0.21 |
| SD1C | 12.99±0.65* | 13.78±0.37 | 12.91±0.54** | 13.24±0.71 |
| SD2A | 12.29±0.29* | 13.37±0.04 | 12.39±0.16** | 13.37±0.38 |
| WYG377 | 10.41±0.86 | 13.55±0.66 | 10.45±0.83 | 13.24±0.61 |
| WYG1A | 12.70±0.15** | 13.49±0.36 | 13.11±0.52** | 13.34±0.69 |
| WYG1C | 12.91±0.48** | 13.23±0.72 | 12.85±0.36** | 13.36±0.28 |
| WYG2A | 12.18±0.14* | 13.39±0.63 | 12.89±0.40** | 13.47±0.17 |

The area of each plot was 13.3 m^2^. Values are the means (± SD) (*n* = 3). *, **, significantly different from the recurrent parent at *P* < 0.05 and *P* < 0.01 respectively.
